# An artificial intelligence-based chatbot as a tool for promoting nutrition in a community setting

**DOI:** 10.1093/oodh/oqag037

**Published:** 2026-09-23

**Authors:** Rajabu Simba, Gilbert Charles, John B Uiso, Jacqueline Wetson, Hyasinta Jaka, Leonard Katalambula

**Affiliations:** Department of Public Health and Community Nursing, University of Dodoma, 1 Benjamin Mkapa Road, Iyumbu, Dodoma District, P.O. Box 259, Dodoma, 41218, The United Republic of Tanzania; Department of Physiology, Muhimbili University of Health and Allied Sciences, 9 United Nations Road, Upanga West, Ilala District, P.O. Box 65001, Dar es Salaam, The United Republic of Tanzania; Department of Nutrition, Muhimbili National Hospital, Kalenga Street, Malik Road, Upanga West, Ilala District, P.O. Box 65000, Dar es Salaam, The United Republic of Tanzania; Department of Public Health and Community Nursing, University of Dodoma, 1 Benjamin Mkapa Road, Iyumbu, Dodoma District, P.O. Box 259, Dodoma, 41218, The United Republic of Tanzania; Department of Internal Medicine, Gastroenterology and Hepatology Unit, Catholic University of Health and Allied Sciences, 35 Wurzburg Road, Bugando, P.O. Box 1464, Mwanza, 33102, The United Republic of Tanzania; Department of Public Health and Community Nursing, University of Dodoma, 1 Benjamin Mkapa Road, Iyumbu, Dodoma District, P.O. Box 259, Dodoma, 41218, The United Republic of Tanzania

**Keywords:** artificial intelligence chatbots, nutrition education, digital health in Tanzania

## Abstract

Malnutrition remains a major public health problem in Tanzania, worsened by limited access to personalized, culturally relevant nutrition information. Existing gaps in community nutrition education across sub-Saharan Africa, combined with conventional approaches ignoring individual dietary needs, contribute to undernutrition and chronic disease. However, while AI-powered chatbots have shown global effectiveness in scalable health education, evidence from low-resource, Swahili-speaking settings is scarce. This study, aligned with Sustainable Development Goals (SDGs) 2, 3, and 4, developed Lishe Yetu, a culturally localized digital nutrition tool aimed at bridging these gaps. A pre-post quasi-experimental mixed-methods design evaluated Lishe Yetu among 400 Tanzanian residents between January and April 2025. Built on retrieval-augmented generation using GPT-3.5-turbo, the platform drew from over 2500 expert-reviewed sources including World Health Organization (WHO), Food and Agriculture Organization of the United Nations (FAO), Tanzania Food and Nutrition Centre, and validated Swahili materials. Nutritional knowledge was measured using a validated 20-item questionnaire, with engagement tracked via server logs and satisfaction assessed through Likert-scale surveys and thematic analysis of qualitative feedback. Results showed strong engagement, with average sessions lasting 12 minutes and 15.2 messages exchanged; 87% completed at least one full session. Satisfaction averaged 4.3/5.0, and 88% found the tool easy to use. Knowledge scores improved significantly from 52% to 73%, a 21 percentage-point gain with a large effect size (Cohen’s d = 1.57, *P* < 0.001). Additionally, 65% reported intentions to adopt healthier diets. These findings provide strong preliminary evidence that a Swahili-language AI chatbot can improve nutrition literacy in low-resource African settings, though randomized controlled trials are needed to confirm sustained behaviour change.

## Introduction

Malnutrition, encompassing both undernutrition and overnutrition, remains a major public health problem in Tanzania, disproportionately affecting children, adolescents, and women of reproductive age (Mduma and Kalegele, 2015; Viljoen et al., 2015). Conventional community nutrition programmes rely on manual, one-size-fits-all education that fails to address individual dietary needs, contributing to the persistence of stunting, anaemia, obesity, and hypertension. Artificial intelligence (AI)-powered chatbots are being used for health communication, including the World Health Organization’s Florence digital health worker (World Health Organization, 2020). Other digital nutrition resources include the United States Department of Agriculture's (USDA) Start Simple with MyPlate mobile app (United States Department of Agriculture, 2020), Australia’s Commonwealth Scientific and Industrial Research Organisation (CSIRO) Healthy Diet Score online assessment (Commonwealth Scientific and Industrial Research Organisation, 2025), and the UK’s NHS Weight Loss Plan app (National Health Service, 2026). These three resources are not identified by their providers as AI nutrition chatbots, and their availability alone does not establish improvements in dietary guidance or adherence. In developing countries such as Tanzania, however, urbanization, changing food systems, and weak health infrastructure have produced a double burden of malnutrition (Mduma and Kalegele, 2015; Viljoen et al., 2015), and empirical evidence on AI chatbot interventions from Swahili-speaking, low-resource settings remains scarce. Local platforms such as Dietech’s Our Nutrition chatbot (Dietech Tanzania, 2025), launched in January 2025 and used by over 12 000 people within its first 4 months, illustrate growing demand for such tools, yet few studies have evaluated chatbot interactivity, personalization, or user engagement within Tanzanian community settings (Brindha et al., 2024; Dutta and Koti, 2023). This gap is significant because existing Tanzanian mobile-health research has largely focused on general applications rather than AI-based, real-time, interactive systems (Mduma and Kalegele, 2015, 2017; Mramba and Kaijage, 2018).

This study therefore evaluates Lishe Yetu (Our Nutrition), a Swahili-language AI chatbot integrated into the Dietech platform, as a tool for promoting nutritional awareness and behaviour change in Tanzanian communities. Specifically, the study examines: (i) the level of user engagement with and satisfaction toward the chatbot; (ii) its effect on users’ understanding of basic nutritional concepts; (iii) its influence on users’ intention to adopt healthier eating behaviours; and (iv) the barriers and opportunities for scaling AI chatbot-based nutrition education within Tanzanian community health systems. In addressing these questions, and aligned with SDGs 2, 3 and 4, the study contributes empirical evidence on AI chatbot interventions in a low-resource, Swahili-speaking context, and offers practical insight for researchers, developers, nutritionists, and health planners seeking to design and scale similar culturally adapted digital health tools.

## Theoretical framework

For this study, the Unified Theory of Acceptance and Use of Technology (UTAUT) is adopted as the guiding theoretical framework. UTAUT integrates elements from eight prior models of technology acceptance and aims to explain user intentions to use a technology and subsequent usage behavior. The model identifies four core determinants of user acceptance and use behavior: performance expectancy, effort expectancy, social influence, and facilitating conditions.

In the context of this research, performance expectancy reflects the belief that the Lishe Yetu chatbot will enhance users’ knowledge and decision-making regarding nutrition. Effort expectancy relates to the perceived ease of use of the chatbot, whether users find it intuitive, clear, and accessible in Swahili. Social influence involves the extent to which users perceive that important others (e.g. peers, health workers, or community leaders) believe they should use the chatbot. Facilitating conditions pertain to the technical and social infrastructure available to support the use of Lishe Yetu, including mobile phone access and digital literacy.

This theory is well-suited to the study because it provides a structured approach for understanding how users engage with health technology in a community setting. UTAUT guided the development of data collection instruments such as surveys and interviews, and shaped the analysis of factors influencing the adoption and impact of Lishe Yetu.

### Empirical literature review

#### Level of user engagement and satisfaction with AI chatbots for nutrition education

Ünal and Çılgın (Ünal and Çilgın, 2022) designed and implemented a web-based expert system for personal nutrition planning and found the tool effective in delivering personalized nutrition plans, noting positive user interaction and satisfaction. Similarly, Benson (Benson, 2023) evaluated AI chatbots in food policy research and found that custom chatbots significantly improved user engagement by simplifying access to policy-related nutrition information.

Dutta and Koti (Dutta and Koti, 2023) developed a recommendation system for personalized nutrition and found high user satisfaction due to accurate and tailored dietary recommendations. These studies collectively demonstrate that users respond positively to personalized AI nutrition platforms, validating the importance of user-focused design in AI-based tools.

#### AI-powered nutrition platforms and users’ knowledge and understanding of nutritional concepts

Héritier et al. (Héritier et al., 2023) investigated personalized nutrition using a digital cohort study and found that the digital platform enhanced nutrition insights by tracking user responses and patterns over time. Rojanaphan (Rojanaphan, 2024) developed a deep learning-based system to detect nutrient deficiencies and found that it significantly improved users’ understanding of their dietary gaps. The International Food Policy Research Institute’s Nudging for Good project (International Food Policy Research Institute, 2025) developed the AI-assisted Food Recognition Assistance and Nudging Insights mobile application for dietary assessment and nutrition-related prompts among adolescent girls in Ghana and Viet Nam. This work evaluated a food-recognition application rather than a conversational nutrition chatbot.

Murakami et al. (Murakami et al., 2021) created a dietary feedback system in Japan which resulted in better comprehension of nutrition among users due to personalized feedback loops. These findings reinforce that AI tools can contribute significantly to enhancing nutrition literacy across demographics.

#### AI chatbots and users’ intention to adopt healthier dietary behaviours

Kalyoncu Atasoy et al. (Kalyoncu Atasoy et al., 2024) studied AI-powered strategies targeting vulnerable groups and found that such interventions resulted in positive changes in nutritional behavior. Similarly, Sadique Abdallah et al. (Abdallah et al., 2024) explored AI tools across South Africa, India, and the USA using deep learning models, and demonstrated improved dietary outcomes in diverse populations.

Viljoen et al. (Viljoen et al., 2015) examined mobile health (mHealth) strategies and concluded that digital health applications improved nutrition outcomes by influencing daily dietary decisions. The Nudging for Good project (International Food Policy Research Institute, 2025) examined an AI-assisted mobile dietary assessment and nudging tool among adolescent girls in Ghana and Viet Nam. Its design and findings should not be interpreted as direct evidence of the effects of a conversational nutrition chatbot. Together, these examples illustrate the range of digital approaches used to support nutrition education and dietary decision-making.

#### System-level and contextual challenges affecting implementation and scalability

Mduma and Kalegele (Mduma and Kalegele, 2015, 2017) identified infrastructural and technological constraints in Tanzania that limited the long-term adoption and scaling of nutrition-focused mobile apps. Similarly, Maina-Gathigi et al. (Maina-Gathigi et al., 2017) and NiPN (Dure Technology, 2019) reported gaps in data accuracy, integration, and usability within Kenya’s national nutrition information systems, highlighting systemic challenges AI tools face in operational environments.

Bälter et al. (Bälter et al., 2022) in Ethiopia and Rwanda observed a disconnect between digital health innovations and on-ground implementation, pointing to a need for stronger alignment between digital tools and local health systems. Yevtushenko et al. (Yevtushenko et al., 2024) echoed this by stressing the lack of real-time surveillance infrastructure in global nutrition monitoring efforts. These findings underscore that for AI-based chatbot interventions to be effective and scalable in community nutrition education, systemic readiness, contextual tailoring, and infrastructural support are critical.

While the reviewed literature affirms the positive role of AI-powered systems in enhancing user engagement, nutrition knowledge, and behavior change, a significant gap exists in their contextual application within Tanzania. Most studies have been conducted in global settings or high-income countries, with few focusing on Swahili-speaking, low-resource communities. Although Mduma and Kalegele (Mduma and Kalegele, 2015, 2017) and Mramba and Kaijage (Mramba and Kaijage, 2018) explored mobile nutrition tools in Tanzania, these did not involve AI-based chatbots or real-time, interactive, personalized systems like Lishe Yetu. This study fills an important gap by empirically assessing the Lishe Yetu AI chatbot in Tanzania, using performance data, user feedback, and nutritional outcomes, thus offering a localized and scalable solution to the malnutrition problem.

### Conceptual framework

This study is guided by a conceptual framework that integrates UTAUT and empirical evidence on AI-driven nutrition interventions. As shown in Fig. 1, the framework illustrates how the Lishe Yetu chatbot is expected to enhance nutritional knowledge and encourage healthier eating behaviors within Tanzanian communities, through four key factors influencing user acceptance: performance expectancy, effort expectancy, social influence, and facilitating conditions, moderated by user engagement and personalization of information.

**Figure 1:**
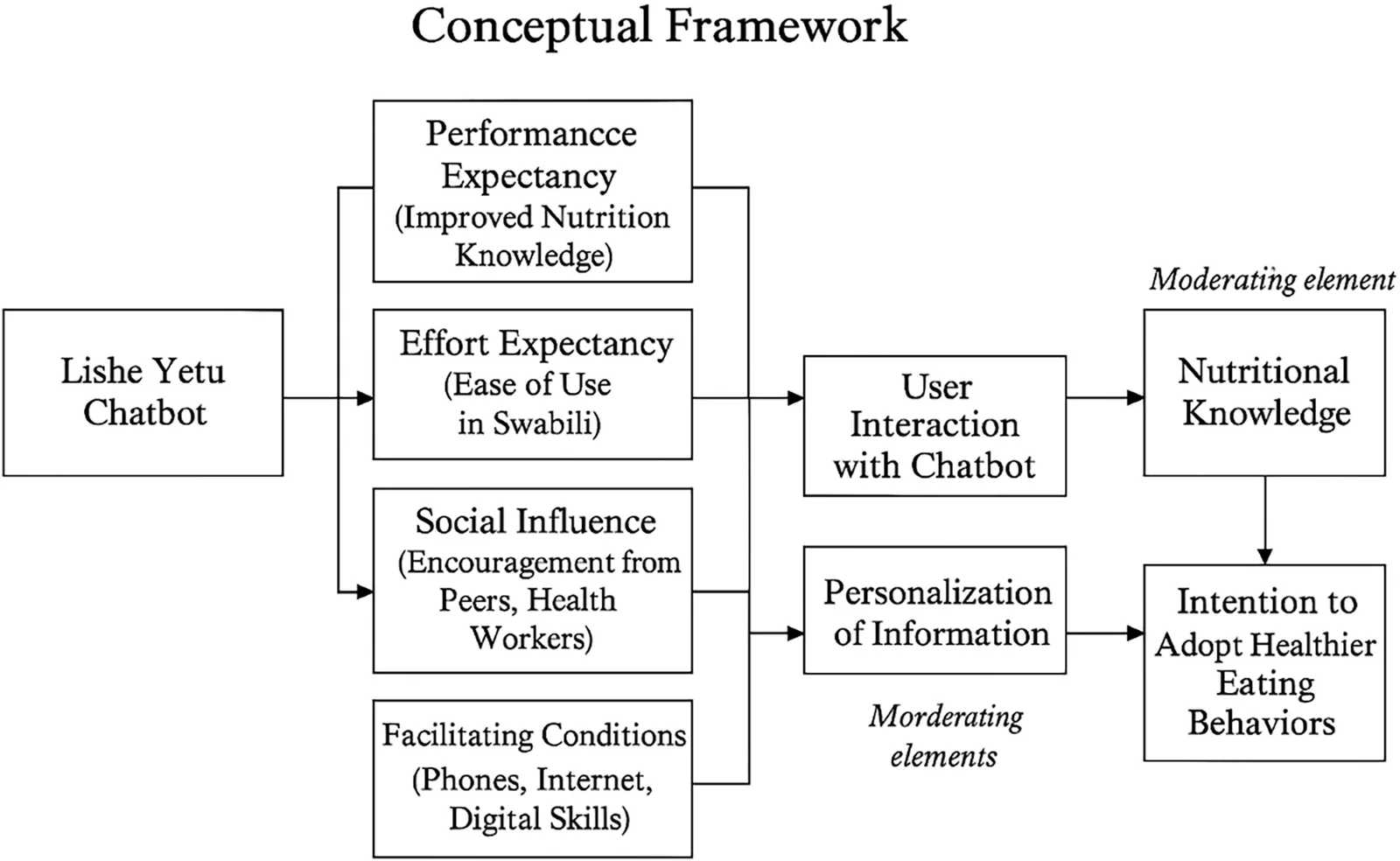
Conceptual framework integrating UTAUT and AI-driven nutrition interventions for the Lishe Yetu chatbot.

## Materials and methods

### Chatbot development

The Our Nutrition AI chatbot was designed and developed using natural language processing (NLP) algorithms. The chatbot’s knowledge base was constructed through training on extensive datasets covering nutritional science, dietary guidelines, and culturally relevant local food practices specific to Tanzanian communities. The chatbot was implemented as a web-based platform, enabling accessible deployment across diverse user groups via internet-enabled devices. Specifically, the chatbot was built on a retrieval-augmented generation (RAG) architecture combining a large language model (LLM) backbone, GPT-3.5-turbo via the OpenAI API, with a curated domain-specific knowledge base. Responses generated by the LLM were constrained to this verified knowledge base to minimize hallucination and ensure factual accuracy. The model was evaluated prior to deployment using a set of 150 expert-validated nutrition questions; accuracy was 91.3% when compared to reference answers reviewed by registered dietitians. To support independent validation, the chatbot’s system prompt, knowledge base schema, and evaluation protocol are available from the corresponding author upon reasonable request.

To optimize user interaction and ensure cultural appropriateness, usability studies were conducted during the development phase. These studies assessed user engagement, accuracy of nutritional information provided, and impact on knowledge retention, informing iterative refinements to the chatbot’s dialogue flows, response clarity, and content relevance.

### Technical architecture and development pipeline

Lishe Yetu was built on a hybrid rule-based and retrieval-augmented NLP pipeline. The system combined a LLM backbone (GPT-3.5-turbo, OpenAI API) with a structured retrieval layer. User queries were first pre-processed by a lightweight intent-classification module implemented in Python (spaCy v3.5) that categorised each query into one of seven nutritional intent classes (nutrient inquiry, meal planning, food substitution, portion guidance, population-specific advice, general wellness, and out-of-scope). Queries classified as in-scope were routed to the retrieval layer, which performed semantic similarity search (cosine similarity using sentence-transformers/paraphrase-multilingual-MiniLM-L12-v2) against the domain knowledge base to retrieve the top-3 most relevant knowledge entries. These entries were then passed as context to the LLM, which generated a Swahili-language response constrained to the retrieved content. Out-of-scope queries triggered a pre-defined redirection response advising the user to consult a healthcare professional. The full pipeline was containerized using Docker and deployed on a Linux-based cloud server (Ubuntu 22.04, 8 vCPU, 16 GB RAM).

### Knowledge Base composition and curation

The knowledge base comprised 2500 structured nutrition entries, each consisting of a question-answer pair reviewed by at least one registered dietitian. Sources included: (i) WHO Healthy Diet factsheets (2020 edition); (ii) Tanzania Food and Nutrition Centre (TFNC) National Nutrition Guidelines (2016); (iii) Tanzania Ministry of Health Food Composition Tables; (iv) FAO/WHO Codex Alimentarius food safety standards; and (v) validated Swahili-language nutrition education materials developed by the Dietech Tanzania platform. All entries were translated into Swahili by two bilingual nutrition professionals and cross-validated for terminological accuracy. The final knowledge base covered balanced diet principles, macronutrient and micronutrient guidance, maternal and child nutrition, management of common nutritional deficiencies (anaemia, vitamin A deficiency, iodine deficiency), and locally relevant food group classifications.

### Pre-deployment validation and response accuracy

Prior to public deployment, the chatbot was evaluated using a structured validation protocol. A set of 150 expert-validated nutrition questions was constructed by two registered dietitians (RD1 and RD2) independently, covering all seven intent classes in proportion to their expected frequency of use. For each question, a reference answer was agreed upon by consensus between RD1 and RD2. The chatbot responses were then independently rated by a third dietitian (RD3) who was blinded to the reference answers, using a three-point scale: Accurate (2), Partially Accurate (1), or Inaccurate (0). Overall accuracy (proportion rated Accurate or Partially Accurate) was 91.3% (137/150). Intent classification accuracy was 89.3% (134/150 correctly routed). Errors were predominantly in the out-of-scope class (eight misclassifications); these were corrected before launch by expanding the out-of-scope keyword list. The validation instrument, question bank, and scoring rubric are available from the corresponding author upon reasonable request.

## Study design

This was a cross-sectional, mixed-methods study grounded in a pragmatist research philosophy, combining quantitative data (surveys and chatbot analytics) with qualitative data (open-ended user feedback) to comprehensively evaluate the effectiveness of the Lishe Yetu chatbot. The study followed a deductive approach, testing hypotheses derived from the UTAUT and prior empirical evidence on AI in health promotion. Data were collected at a single point in time to assess user interaction with the chatbot, measure knowledge change, and evaluate behavioral intentions, appropriate for capturing immediate outcomes of chatbot use across a diverse user base.

### Study area

The study was conducted in Tanzania, focusing on both urban and rural communities across regions with varying socio-economic backgrounds. The chatbot was accessible via the Dietech platform’s website and promoted nationally, enabling broad reach and diversity in user demographics. According to the study authors, the study-period website was https://www.dietech.greatsite.net; the current Dietech website is https://dietech.or.tz/ (Dietech Tanzania, 2025).

### Population and sampling

The target population comprised Tanzanian residents aged 18 years and above who interacted with the Our Nutrition chatbot between January and April 2025, including students, parents, professionals, and older adults. A non-probability purposive sampling method was used to select participants who actively used the chatbot and completed both the pre- and post-interaction surveys. A total of 400 participants met these criteria and were included in the analysis, based on the completeness of their interaction logs and survey responses. Participant demographic characteristics are reported in the Results section (Table 1).

**Table 1:** Participants’ demographic characteristics.

Variable	Category	Frequency (n)	Percentage (%)
Age group (years)	18–25	142	35.5
	26–35	92	23.0
	36–45	74	18.5
	46–60	60	15.0
	>60	32	8.0
Sex	Female	217	54.3
	Male	183	45.7
Location	Urban	252	63.0
	Peri-urban/Rural	148	37.0
Occupation	Student	164	41.0
	Professional	140	35.0
	Self-employed	64	16.0
	Retired/Older adult	32	8.0
Education level	Primary	28	7.0
	Secondary	116	29.0
	Tertiary/University	256	64.0

### Data collection methods

This study employed a combination of data collection methods to capture both quantitative and qualitative insights on user interaction with the Our Nutrition AI chatbot. Online surveys were the primary tool used to measure changes in participants’ nutritional knowledge and behavioral intentions, with structured questionnaires administered both before and after chatbot interaction. To ensure comparability between pre- and post-intervention assessments, both instruments consisted of identical 20-item multiple-choice questions drawn from the same validated item bank covering four nutritional domains: balanced diets (five items), micronutrients (five items), hydration (five items), and food groups (five items). Items were randomized in order across the two administrations to reduce recall bias.

The pre-survey was completed before the user’s first chatbot session, and the post-survey was administered upon session completion, minimizing the time interval between exposure and assessment.

Engagement metrics were derived from automatically captured server logs. Session frequency was defined as the number of distinct chatbot sessions per user. Average session duration was calculated as the total time (in minutes) from session initiation to final user message. Messages per session were counted as all user-initiated and system-generated exchanges within a single session window. These definitions were applied consistently across all 400 user logs.

### Operational definitions of engagement metrics

The following definitions were pre-specified and applied uniformly: (i) Session – a continuous user-chatbot interaction delimited by a 30-minute inactivity timeout; (ii) Message – any single text submission by the user (excluding system prompts and navigation buttons); (iii) Session completion – a session reaching at least the fourth dialogue turn with a natural or explicit close, determined via dialogue-state tracking; (iv) Session duration – elapsed time (minutes) from the first user message to the last system response within a session; (v) Engagement index – a composite calculated as (messages/session × session duration)/10, used to rank engagement depth. All metrics were extracted from server-side logs by a single analyst using a pre-written Python extraction script.

### Rationale for engagement metric definitions and thresholds

These operational definitions including the 30-minute inactivity timeout for delimiting sessions, the four-dialogue-turn criterion for session completion, and the composite engagement index formula were developed specifically for this study and were not adapted from a previously validated instrument. The 30-minute timeout follows a threshold commonly applied in web-analytics practice to distinguish a genuine break in interaction from ordinary response latency. The four-turn threshold was chosen as the minimum number of exchanges considered necessary for a user to receive a substantive nutrition response rather than a superficial query. The engagement index formula was constructed post hoc to allow relative ranking of engagement depth across users. As these thresholds and the composite index have not been independently validated against an external criterion, we report them here as study-specific methodological choices and acknowledge, as a limitation, that they require validation in future research before being adopted as standard measures.

In addition to survey data, chatbot analytics were utilized to collect automated usage data, including frequency and duration of interactions, navigation paths, and user engagement patterns. Optional feedback forms integrated into the platform allowed users to share perceptions regarding usability, cultural relevance, and overall experience. Follow-up surveys evaluated short-term behavioral intentions and early signs of dietary behavior change post-intervention.

### Data analysis

Data analysis in this study employed a multi-faceted approach that integrated quantitative, qualitative, and sentiment analysis techniques to comprehensively evaluate the impact of the Our Nutrition AI chatbot. Quantitative data, such as nutritional knowledge scores, user satisfaction ratings, and behavioral intention measures, were analysed using descriptive statistics including means, frequencies, and percentages, consistent with methodologies outlined by Creswell and Creswell (Creswell and Creswell, 2018). Inferential statistical tests, specifically paired sample t-tests and chi-square tests, were conducted using Python’s scientific libraries to determine significant differences in nutritional knowledge and behavioral intentions before and after chatbot interaction, providing an objective assessment of the chatbot’s educational and motivational effects (Field, 2018).

Server logs automatically captured chatbot interaction data, including session frequency, average duration, and navigation patterns. These usage metrics were analysed through frequency counts and pattern recognition techniques implemented in pandas, allowing identification of user engagement levels and common interaction flows. Visualization of these patterns was performed with matplotlib in Jupyter Notebook, enabling clear graphical representation of engagement trends and behavior over time (Silverman, 2016).

Qualitative data, gathered from open-ended survey questions and user feedback forms, were exported to Microsoft Excel for organization and initial coding. Thematic analysis was performed following Braun and Clarke’s framework (Braun and Clarke, 2006), involving iterative categorization of responses to identify dominant themes related to usability, cultural relevance, content accuracy, and perceived behavior change. Complementing thematic coding, sentiment analysis was applied to qualitative comments using automated sentiment scoring tools within Python, classifying responses into positive, neutral, or negative categories (Liu, 2012). The combined use of quantitative, qualitative, and sentiment analyses ensured a robust and nuanced understanding of the chatbot’s performance and user experience.

### Validity and reliability

Validity was ensured by developing survey instruments in consultation with nutrition and public health experts, aligning with study objectives. Construct validity was supported by grounding the survey design in the UTAUT theoretical framework. Reliability testing of survey instruments using Cronbach’s alpha yielded acceptable internal consistency (α ≥ 0.70). A pilot test involving a small group of users assessed clarity, usability, and technical functionality, informing final revisions.

## Results

### Participant characteristics

Participant demographic characteristics were as follows: the mean age was 31.4 years (SD = 10.2; range: 18–72 years), with the majority falling in the 18–35 age group (58.5%). Females comprised 54.3% of respondents. Regarding geographic distribution, 63% of participants were from urban settings (primarily Dar es Salaam, Dodoma, and Arusha), while 37% resided in peri-urban or rural areas. Occupational categories included students (41%), employed professionals (35%), self-employed individuals (16%), and retirees or older adults (8%). A summary is presented in Table 1. These demographics reflect a relatively digitally connected sample, consistent with the chatbot’s web-based delivery mode, and are reported to allow readers to assess the representativeness and generalizability of the findings.

### User engagement and satisfaction

User engagement with the Our Nutrition chatbot was quantitatively assessed by analyzing server logs that recorded interaction frequency, average session duration, and the number of messages exchanged per session. User satisfaction was evaluated through post-interaction online surveys employing Likert-scale questions focused on the chatbot’s informativeness, ease of use, and overall user experience. A summary of these key metrics is presented in Table 2.

**Table 2:** User engagement and satisfaction metrics.

Metric	Value	Percentage (%)
Completed full session	348	87
Average messages per session	15.2	-
Average session duration (min)	12	-
Rated informative/very informative	-	85.5
Rated easy to use	-	88
Mean satisfaction score (1–5)	4.3 ± 0.6	-

On average, each session involved 15.2 messages, demonstrating active interaction rather than passive usage. Sessions lasted ~ 12 minutes, and 87% of users completed at least one full interaction session. A substantial 85.5% of respondents rated the chatbot as either ‘informative’ or ‘very informative’, while 88% found it easy to use. The mean satisfaction score on a 5-point scale was 4.3 (SD = 0.6). The distribution of satisfaction ratings is illustrated in Fig. 2.

**Figure 2:**
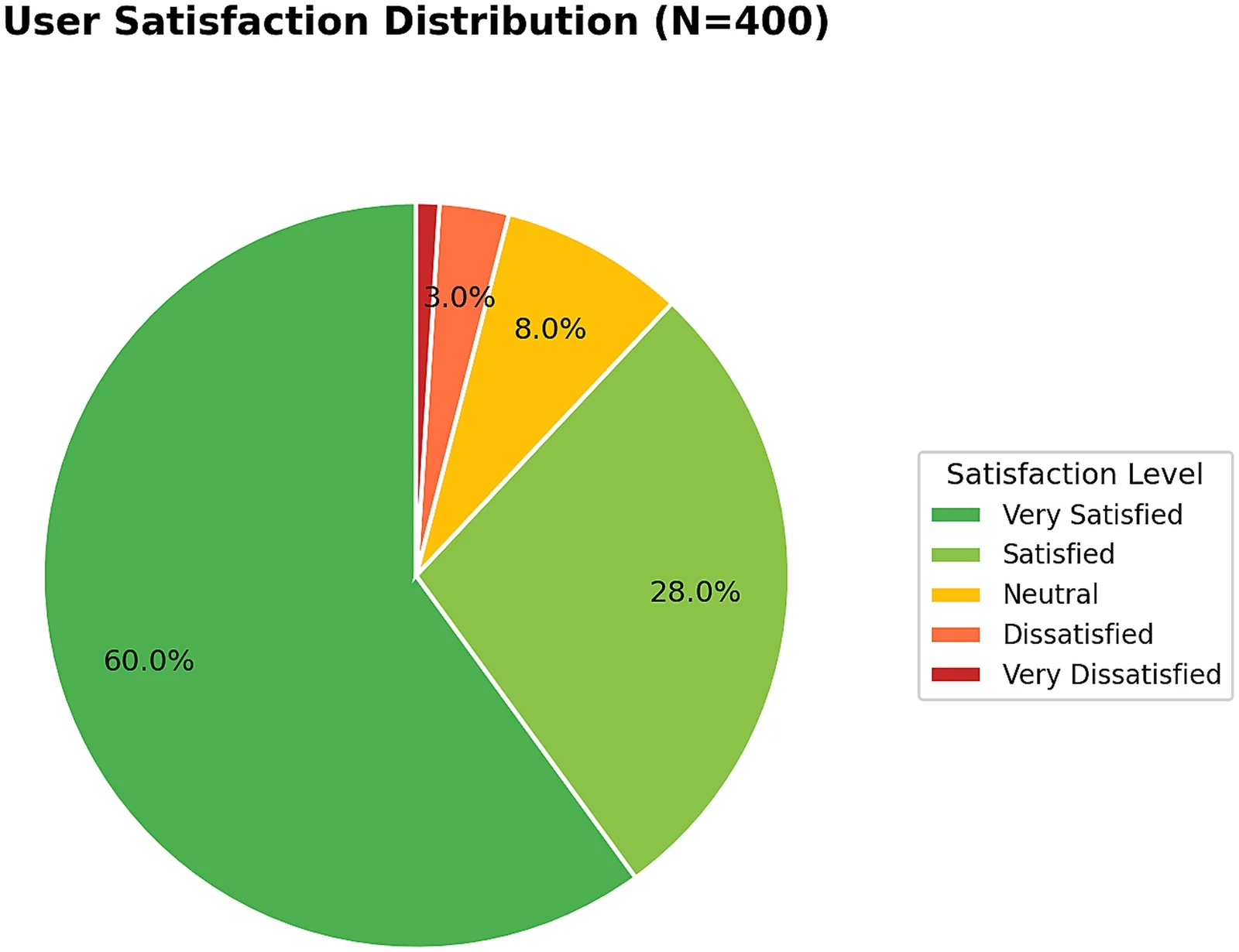
User satisfaction distribution.

### Improvement in nutritional knowledge

The effectiveness of the chatbot in enhancing users’ understanding of key nutritional concepts was evaluated using pre- and post-intervention quizzes assessing knowledge on balanced diets, micronutrients, hydration, and food groups. Prior to interacting with the chatbot, users had a mean knowledge score of 52% (±14%). Following engagement with the chatbot, this score increased significantly to 73% (±12%), a mean improvement of 21 percentage points, equivalent to a 40% relative improvement. A paired *t-*test confirmed that this change was statistically significant, t(399) = 18.25, *P* < 0.001 (Table 3).

**Table 3:** Nutritional knowledge scores pre- and post-intervention.

Metric	Score (%)	Standard Deviation (±)
Mean pre-intervention score	52	14
Mean post-intervention score	73	12
Mean improvement	21 (↑40%)	–
Paired *t-*test	t(399) = 18.25	p < 0.001

### Influence on intentions to adopt healthier dietary practices

Post-interaction survey responses indicated that 65% of users intended to change their dietary behaviors based on what they learned from the chatbot, 20% were unsure, and 15% had no intention to change (Fig. 3). Frequently asked questions revealed common themes around healthy meal planning, portion control, and food substitutions. Analysis of chatbot interaction logs, with main thematic areas detailed in Table 4, highlighted key thematic areas aligned with the study’s objectives.

**Figure 3:**
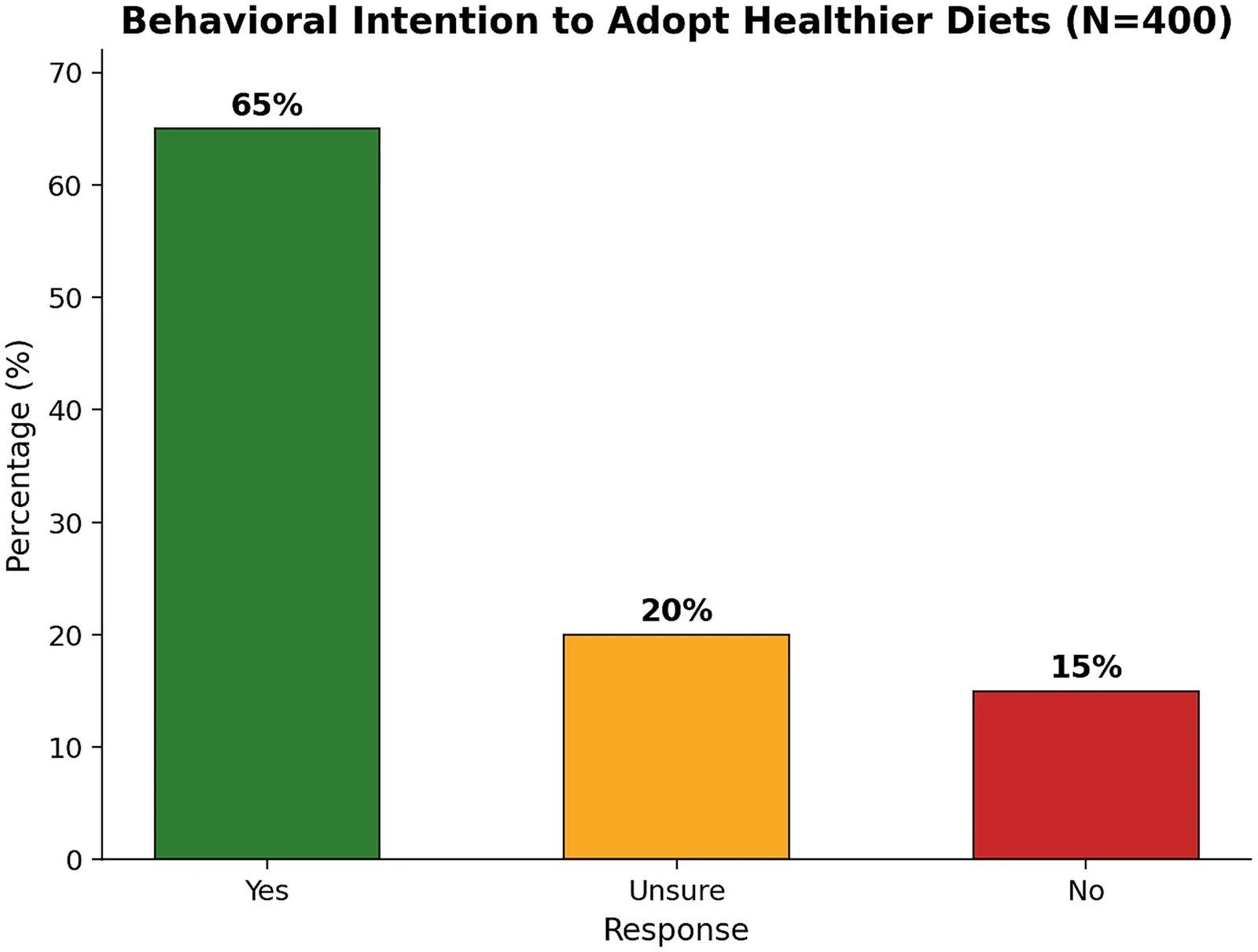
Behavioral intention to adopt healthier diets.

**Table 4:** Main themes of user questions in the chatbot.

Main themes/topics asked	Example questions	Related objective(s)	Implication
Basic nutritional concepts	‘What are the food groups?’, ‘Why is protein important?’	Knowledge improvement	Indicates users sought foundational knowledge, aligning with observed knowledge gains.
Healthy eating practices and meal planning	‘How to plan a balanced meal?’, ‘What are healthy snacks?’	Behavioral intention	Reflects user interest in applying nutrition knowledge to daily life.
Local and cultural food recommendations	‘What are healthy traditional foods?’, ‘How to prepare vegetables?’	Engagement & satisfaction	Enhances cultural relevance and engagement by addressing local dietary habits.
Nutritional advice for specific groups	‘What should pregnant women eat?’, ‘Nutrition for children?’	Knowledge & behaviour	Highlights chatbot’s role in delivering personalized advice.
Addressing malnutrition and deficiencies	‘How to prevent anaemia?’, ‘Signs of vitamin deficiency?’	Knowledge improvement	Shows user interest in addressing real-world nutrition concerns.
Practical tips for dietary change	‘How to reduce sugar intake?’, ‘Best alternatives to fried foods?’	Behavioral intention	Indicates actionable intentions to adopt healthier dietary habits.
Technical and usability questions	‘How to use the chatbot?’, ‘Is my data safe?’	Engagement & satisfaction	Reflects importance of usability and trust in sustaining chatbot use.

### Challenges and opportunities

Server-log and feedback analysis identified key system-level challenges and opportunities for scaling AI chatbot-based nutrition education, summarized in Table 5: limited internet access in rural areas, digital literacy gaps especially among elderly users, and occasional response delays due to server load, set against high smartphone penetration in urban areas, strong cultural and linguistic relevance, and integration possibilities with mHealth platforms and local health services.

**Table 5:** System-level challenges and opportunities.

Aspect	Challenges	Opportunities
Infrastructure	Poor rural internet coverage	Urban smartphone access
User capability	Low digital literacy among some demographics	Community training and support
Technical	Server response delays	Future mobile app integration
Content	Need for ongoing updates to reflect local foods	Strong local cultural adaptation

## Discussion

The high level of user engagement and satisfaction observed demonstrates the chatbot’s success in meeting the needs of Tanzanian users. These findings align closely with previous studies emphasizing the importance of user-friendly, culturally relevant AI interfaces in nutrition education: Ünal and Çılgın (Ünal and Çilgın, 2022) demonstrated that personalized nutrition systems with intuitive designs encourage better engagement, Benson (Benson, 2023) similarly highlighted the role of tailored content in enhancing user satisfaction, and the observed interaction metrics echo Dutta and Koti’s (Dutta and Koti, 2023) findings that AI nutrition tools can effectively engage diverse user groups. The combination of NLP capabilities with culturally tailored nutritional content likely contributed to the chatbot’s appeal and usability.

The 40% relative improvement in nutritional knowledge confirms that the chatbot effectively enhanced nutritional literacy, consistent with findings from Héritier et al. (Héritier et al., 2023) and Murakami et al. (Murakami et al., 2021), who showed AI platforms’ ability to boost understanding through interactive, context-sensitive feedback. The use of culturally appropriate and locally relevant Swahili content likely contributed to improved comprehension and retention among users, especially those from underserved or rural communities, addressing a gap highlighted by Mduma and Kalegele (Mduma and Kalegele, 2015) regarding accessible, tailored nutrition information in Tanzania’s community health initiatives.

The strong behavioral intention shift observed (65% intending to change dietary behaviour) is consistent with previous research by Kalyoncu Atasoy et al. (Kalyoncu Atasoy et al., 2024) and Sadique Abdallah et al. (Abdallah et al., 2024), who found that AI-driven interventions can positively influence health-related decisions, and supports earlier conclusions by Viljoen et al. (Viljoen et al., 2015) about the role of digital nudges in encouraging healthier dietary patterns. User inquiries related to local diets and specific population groups further affirm the chatbot’s contextual and cultural relevance, a factor also highlighted in Tanzanian studies such as Mduma and Kalegele (Mduma and Kalegele, 2015). Overall, the chatbot appears to serve as a relevant, trusted, and impactful digital health tool in supporting nutrition behavior change.

The identified challenges infrastructural and digital-literacy barriers restricting reach in rural and older populations align with documented digital divides in Tanzania (Mramba and Kaijage, 2018). Nevertheless, the culturally tailored design and positive adoption in urban areas point to strong opportunities for expanding AI nutrition education tools through investment in infrastructure and digital literacy training, supporting similar recommendations by Brindha et al. (Brindha et al., 2024).

## Limitations

Several limitations of this study should be acknowledged. First, the study employed a cross-sectional design and measured behavioral intentions rather than actual dietary behavior change; intention-behavior gaps are well-documented in health behavior literature, and future longitudinal studies are required to determine whether the observed knowledge gains and stated intentions translate into sustained dietary improvements. Second, the study relied on a purposive, non-probability sample of participants who self-selected into the Dietech platform, which introduces selection bias; the sample skewed toward urban, more educated, and digitally literate users, limiting generalizability to rural populations and users with lower digital literacy. Third, all outcome measures (nutritional knowledge, behavioral intention, satisfaction) were based on self-reported data, which is susceptible to social desirability bias. Fourth, no control group was included, so the observed pre-to-post knowledge improvement cannot be unambiguously attributed to the chatbot alone; a randomized controlled design would strengthen causal inference in future research. Fifth, the study covered only a four-month period (January–April 2025), which may not capture longer-term patterns of engagement or knowledge decay. These limitations should be considered when interpreting the findings and designing future studies.

## Conclusion

The evaluation of Lishe Yetu demonstrates that AI chatbots can be powerful tools for improving nutritional awareness and encouraging healthier dietary behaviors in Tanzanian communities. The chatbot achieved high user engagement and satisfaction, largely due to its culturally tailored content delivered in Swahili and its user-friendly interface. Users showed significant improvements in nutritional knowledge, with a 40% relative increase after interacting with the chatbot. Furthermore, a majority expressed intentions to adopt healthier diets, indicating a positive influence on behavior change. However, the study also revealed challenges such as limited internet access in rural areas, digital literacy gaps among older users, and occasional technical delays that may hinder widespread adoption. It is important to acknowledge that this study measured behavioral intentions rather than actual long-term dietary behavior change; future longitudinal studies are needed to determine whether the knowledge gains and expressed intentions observed here translate into sustained dietary improvements over weeks or months.

Regarding the reliability and safety of chatbot recommendations, several safeguards were implemented. All nutrition content was verified against TFNC and WHO guidelines prior to deployment. The system was constrained to respond only within its validated knowledge base, and responses flagged as out-of-scope (e.g. medical diagnoses or therapeutic diets for specific conditions) were programmatically redirected to advise users to consult a healthcare professional. Content accuracy was validated by two registered dietitians who reviewed 150 sample responses and rated 91.3% as accurate and appropriate. Notwithstanding these measures, AI-generated responses may contain errors, and the chatbot should not substitute for individualized clinical nutrition counseling.

## Recommendations

To maximize the potential of Lishe Yetu and similar AI-driven nutrition education tools, several key recommendations are proposed. First, expanding internet infrastructure and improving digital literacy, especially in rural and older populations, are critical steps to ensure equitable access; community-based training programs can help bridge the digital divide. Second, developing a dedicated mobile application could enhance performance by reducing server delays and enabling offline functionality. Third, regular updates and localization of nutritional content are essential to maintain cultural relevance and user trust. Fourth, fostering partnerships with local health services and integrating the chatbot into existing mHealth initiatives will increase dissemination and reinforce health education efforts. Finally, conducting longitudinal research to monitor sustained behavior change and health outcomes will provide valuable evidence to guide future improvements.

## Data Availability

The data supporting the findings of this study are available from the corresponding author upon reasonable request. The datasets generated and analysed during the current study are not publicly available due to privacy and ethical restrictions, but can be provided to researchers upon request, with due consideration of ethical guidelines and confidentiality agreements.
